# Metagenome-assembled genomes from time-series samples of artificial seawater aquarium water

**DOI:** 10.1128/mra.00695-26

**Published:** 2026-07-23

**Authors:** Kaho Mori, Yosuke Nishimura, Minoru Ijichi, Yuka Iwahashi, Satoshi Sudo, Susumu Yoshizawa

**Affiliations:** 1 Graduate School of Frontier Sciences, The University of Tokyo, Chiba, Japan; 2 Atmosphere and Ocean Research Institute, The University of Tokyo, Chiba, Japan; 3 Institute for Extra-cutting-edge Science and Technology Avant-garde Research of Life (X-star), Japan Agency for Marine-Earth Science and Technology (JAMSTEC), Kanagawa, Japan; 4 Graduate School of Sciences, Tokyo Metropolitan University 12944, Hachioji, Tokyo, Japan; 5 SEA LIFE Nagoya, Aichi, Japan; Montana State University, Bozeman, Montana, USA

**Keywords:** metagenomics, metagenome-assembled genomes, aquarium, artificial seawater

## Abstract

We report 804 metagenome-assembled genomes (MAGs) reconstructed from a water conditioning tank during the establishment of an artificial seawater aquarium at SEA LIFE Nagoya. These MAGs were assigned to 27 phyla (26 bacterial phyla and 1 archaeal phylum), providing a genome-resolved resource for investigating the microbial diversity of artificially managed marine environments.

## ANNOUNCEMENT

Closed artificial seawater systems, including aquaria and recirculating aquaculture systems, maintain complex microbial communities that play critical roles in nitrogen cycling, water quality stabilization, and the health of reared organisms (1, 2). Despite their functional importance, the genomic diversity of microorganisms in these systems, particularly those established using artificial seawater rather than natural inocula, remains poorly characterized at the genome-resolved level (3). We previously monitored microbial community succession in the water conditioning tank of SEA LIFE Nagoya Aquarium over a 4-month setup period using 16S rRNA gene amplicon sequencing (K. Mori, Y. Sugai, Y. Tsukamoto, Y. Nishimura, M. Ijichi, Y. Iwahashi, S. Sudo, S. Yoshizawa, submitted for publication), revealing increased diversity, dynamic amplicon sequence variant (ASV) turnover, and the establishment of nitrogen removal capacity. To complement these findings with genome-resolved insights, we performed shotgun metagenomic sequencing of 12 samples and reconstructed metagenome-assembled genomes (MAGs).

Water samples were collected from the surface of a water conditioning tank at SEA LIFE Nagoya (Aichi, Japan; 35.0504°N, 136.8463°E) between December 7, 2017, and April 7, 2018, as previously described (K. Mori, Y. Sugai, Y. Tsukamoto, Y. Nishimura, M. Ijichi, Y. Iwahashi, S. Sudo, S. Yoshizawa, submitted for publication). During each sampling event, 2 L of artificial seawater was filtered through Sterivex cartridge filters (0.22 μm pore size; Merck), yielding 43 filters in total. Filters were stored at −18°C immediately after collection until DNA extraction. Twelve filters were selected for metagenomic sequencing. DNA was extracted using the ChargeSwitch Forensic DNA Purification Kit (Thermo Fisher Scientific), and metagenomic libraries were prepared using the MGIEasy FS DNA Library Prep Set (MGI Tech), both following the manufacturer’s instructions. Libraries were sequenced on a DNBSEQ-G400 platform with 150-bp paired-end chemistry, generating 2,903,721,012 raw reads.

Raw reads were quality-filtered and adapter-trimmed using fastp v0.23.4 (4), with parameters --qualified_quality_phred 30, --unqualified_percent_limit 20, --n_base_limit 2, and --detect_adapter_for_pe, resulting in 2,359,106,932 reads. Metagenome assembly was performed using SPAdes v4.1.0 (5), with the --meta option, yielding 550,630–4,319,624 contigs per sample, with N50 values of 469–1,240 bp (see Supplementary data sets at https://doi.org/10.5281/zenodo.21127192). Contig binning was conducted with default options using MetaBAT2 v2.15.15 (6), MaxBin v2.2.7 (7), and CONCOCT v1.1.0 (8). Bins were refined using the bin_refinement module of metaWRAP v1.3.2 (9) with parameters -c 50 and -x 10. Genome completeness and contamination were estimated using CheckM2 v1.0.2 (10), and taxonomic classification was assigned using GTDB-Tk v2.6.1 (11) with the R226 database. Gene prediction and annotation were performed using Prokka v1.14.6 (12) with the --metagenome option.

**Fig 1:**
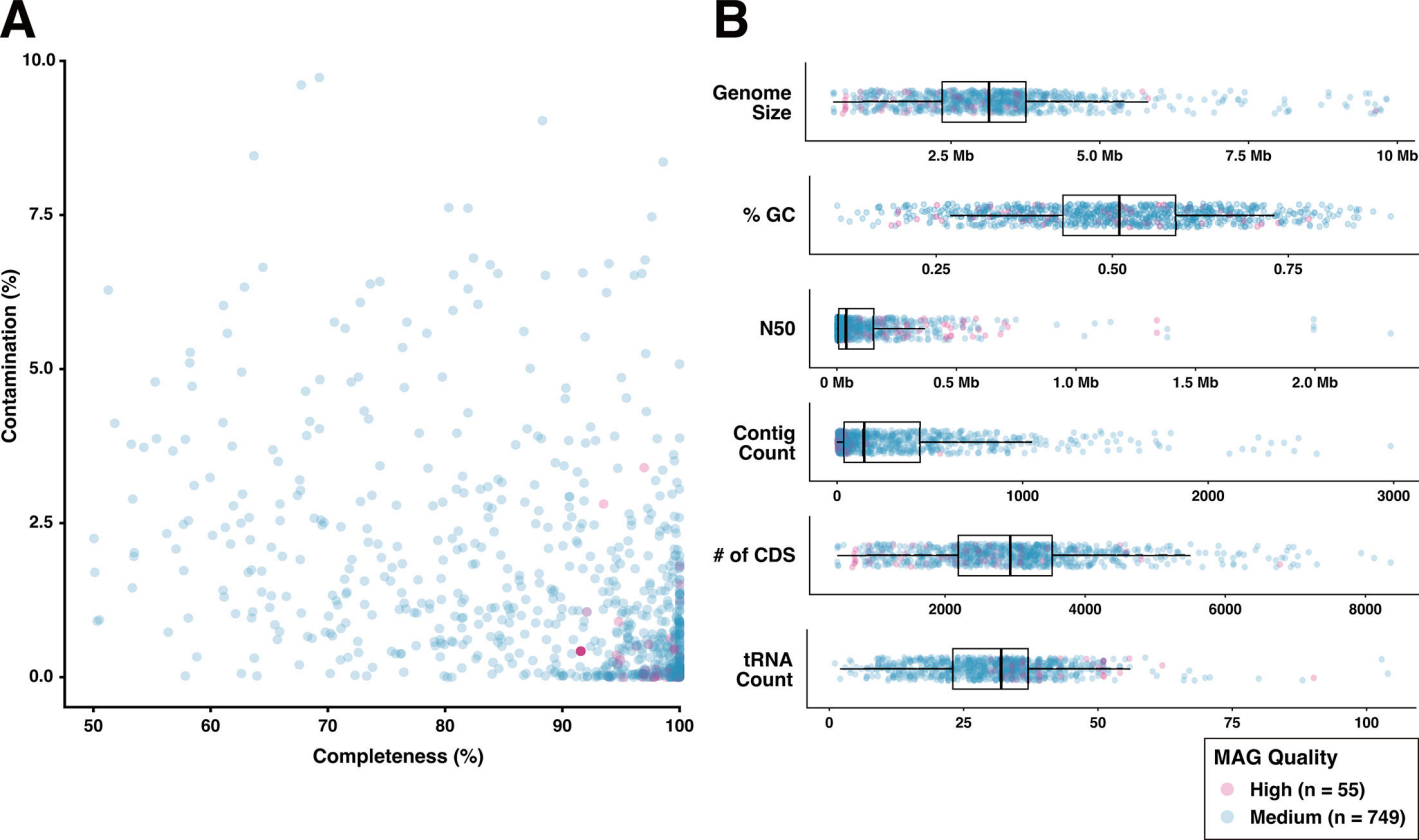
Quality and characteristics of 804 MAGs recovered from the water conditioning tank of SEA LIFE Nagoya. (**A**) Scatter plot of completeness versus contamination for all MAGs, estimated by CheckM2. Colors indicate MAG quality: medium-quality MAGs (blue) and high-quality MAGs (pink), as defined by Bowers et al. (13). (**B**) Distribution of genomic statistics across all MAGs, including genome size (Mb), GC content (%), N50 (bp), number of contigs, number of predicted coding sequences (CDS), and number of tRNAs. Colors correspond to MAG quality as in panel **A**.

Based on the criteria proposed by Bowers et al. (13), we identified 55 high-quality and 749 medium-quality MAGs (Fig. 1A). In total, 804 MAGs were recovered and classified into 27 phyla (26 bacterial phyla and 1 archaeal phylum). Based on CheckM2 predictions, genome sizes ranged from 0.52 to 9.82 Mb, with GC contents of 27%–73% (Fig. 1B). Contig numbers ranged from 1 to 2,984, with N50 values of 1.4 kb to 2.3 Mb. Prokka predicted 461–8,361 coding sequences and 1–104 tRNAs. The reconstructed MAGs encompassed a taxonomically and genomically diverse prokaryotic assemblage (see Supplementary data sets at https://doi.org/10.5281/zenodo.21127192). These MAGs provide a genome-resolved view of microbial community dynamics during the initial establishment of a closed artificial seawater aquarium.

## Data Availability

The raw sequencing reads and individual medium- and high-quality MAG sequences were deposited at DDBJ under BioProject accession number PRJDB42066. The raw sequencing reads are available under SRA accessions DRR959873–DRR959884 (BioSample accessions SAMD01899453–SAMD01899464), and the MAG sequences are available under SRA accessions DRZ139347–DRZ140150 (BioSample accessions SAMD01908863–SAMD01909666). Supplementary data sets containing MAG metadata, Prokka gene annotation results, and SPAdes assembly statistics are available on Zenodo at https://doi.org/10.5281/zenodo.21127192.
